# Comparison of Efficacy of Lokomat and Wearable Exoskeleton-Assisted Gait Training in People With Spinal Cord Injury: A Systematic Review and Network Meta-Analysis

**DOI:** 10.3389/fneur.2022.772660

**Published:** 2022-04-13

**Authors:** Lingjie Zhang, Fabin Lin, Lei Sun, Chunmei Chen

**Affiliations:** ^1^School of Health, Fujian Medical University, Fuzhou, China; ^2^Department of Neurosurgery, Fujian Medical University Union Hospital, Fuzhou, China

**Keywords:** EAW, Lokomat, spinal cord injuries, locomotor, network meta-analysis

## Abstract

**Objective:**

Lokomat and wearable exoskeleton-assisted walking (EAW) have not been directly compared previously. To conduct a network meta-analysis of randomized and non-randomized controlled trials to assess locomotor abilities achieved with two different types of robotic-assisted gait training (RAGT) program in persons with spinal cord injury (SCI).

**Methods:**

Three electronic databases, namely, PubMed, Embase, and the Cochrane Library, were systematically searched for randomized and non-randomized controlled trials published before August 2021, which assessed locomotor abilities after RAGT.

**Results:**

Of 319 studies identified for this review, 12 studies were eligible and included in our analysis. Studies from 2013 to 2021 were covered and contained 353 valid data points (N-353) on patients with SCI receiving wearable EWA and Lokomat training. In the case of wearable EAW, the 10-m walk test (10-MWT) distance and speed scores significantly increased [distance: 0.85 (95% CI = 0.35, 1.34); speed: −1.76 (95% CI = −2.79, −0.73)]. The 6-min walk test (6-MWT) distance [−1.39 (95% CI = −2.01, −0.77)] and the timed up and go (TUG) test significantly increased [(1.19 (95% CI = 0.74, 1.64)], but no significant difference was observed in the walking index for spinal cord injury (WISCI-II) [−0.33 (95% CI = −0.79, 0.13)]. Among the patients using Lokomat, the 10-MWT-distance score significantly increased [−0.08 (95% CI = −0.14, −0.03)] and a significant increase in the WISCI-II was found [1.77 (95% CI = 0.23, 3.31)]. The result of network meta-analysis showed that the probability of wearable EAW to rank first and that of Lokomat to rank second was 89 and 47%, respectively, in the 10-MWT speed score, while that of Lokomat to rank first and wearable EAW to rank second was 73 and 63% in the WISCI-II scores.

**Conclusion:**

Lokomat and wearable EAW had effects on the performance of locomotion abilities, namely, distance, speed, and function. Wearable EAW might lead to better outcomes in walking speed compared with that in the case of Lokomat.

## Introduction

Physical limitations following a spinal cord injury (SCI) can lead to adverse consequences related to motor–autonomic–sensory function, cardiovascular function (1), and bowel function, among others. Moreover, they increase the risk of paralysis, such as muscle atrophy, pressure ulcer, and osteoporosis (2). A decline in physical function increases the need for assistance to be able to perform activities of daily living (ADLs). It also reduces the quality of life for individuals with SCI (3).

There has been an intense technological development of robot-assisted gait training in recent years and patients with SCI can benefit from the recovery of walking (4, 5). Robot-assisted gait training is generally divided into the two types of robots: grounded exoskeletons and wearable exoskeletons. Grounded exoskeletons contain 2 bilateral programmable and actuated robotic joints attached to the patients' legs to facilitate hip and knee movements as they walk on a treadmill with a harness-supported bodyweight system (6). Lokomat is a typically grounded exoskeleton that needs to be used on a treadmill with partial body weight support which is a stationary walking system (7).

In contrast, wearable exoskeletons are designed to support patients with SCI to re-learn standing, weight shifting, and stepping patterns for walking, and can also utilize different environments for training, including flat indoor surfaces, walking outdoors, navigating obstacles, climbing and descending stairs, and performing activities of daily living (4, 8, 9).

Wearable exoskeleton-assisted walking (EAW) is an overground walking system that employs the use of a rigid external frame for holding the lower extremities and trunk and provides power for hip and knee joint movement. Wearable exoskeletons have FDA approval and/or CE mark and are commercially available, namely, Ekso, HAL, Indego, REX, ReWalk, and SMA (10).

Clinical outcomes of SCI depend on the severity and location of the lesion and may involve partial or complete loss of sensory and/or motor function below the level of injury. Lower thoracic lesions cause paraplegia, whereas lesions at the cervical level are associated with quadriplegia (11). Robotic exoskeletons provide an option for mobility for patients with SCI, the neurological level of which ranges from cervical 1 to lumbar 5. Patients with SCI need to select the optimal exoskeleton by considering their residual motor function and severity of spasticity owing to the robot's different structures (9).

Researchers have been increasingly focusing on robotic gait rehabilitation since the intense development of a robotic device. Certain earlier reviews (12, 13) compiled the available evidence on robot-assisted gait training (RAGT); however, firm conclusions could not be drawn due to insufficient evidence owing to the heterogeneity of the studies, small samples, and identified limitations of the trials. Earlier studies used gait velocity as a measure of overall motor capacity and gait recovery. Aguirre-Güemez et al. (12) claimed that gait training in a robotic orthosis had positive effects only on gait performance, strength, and functioning, but none on speed. However, according to the latest review (9), 10-m walk test (10-MWT) and 6-min walk test (6-MWT) are still the most common parameters for evaluation in patients with SCI, as an increasing number of studies have proved that RAGT improves the walking function. But, data that determined the best type of RAGT for improving locomotor ability outcomes in patients with SCI were lacking. Besides, the literature comparing overground wearable exoskeletons with other types of gait therapies is still scarce, especially among patients with SCI. As indicators of locomotor ability are valuable clinical parameters reflecting physical ability and the technology still remains relatively new, we performed a network meta-analysis of randomized and non-randomized controlled trials to assess the clinical effects of two different types of RAGT in patients with SCI.

## Methods

### Search Strategy

**Figure 1:**
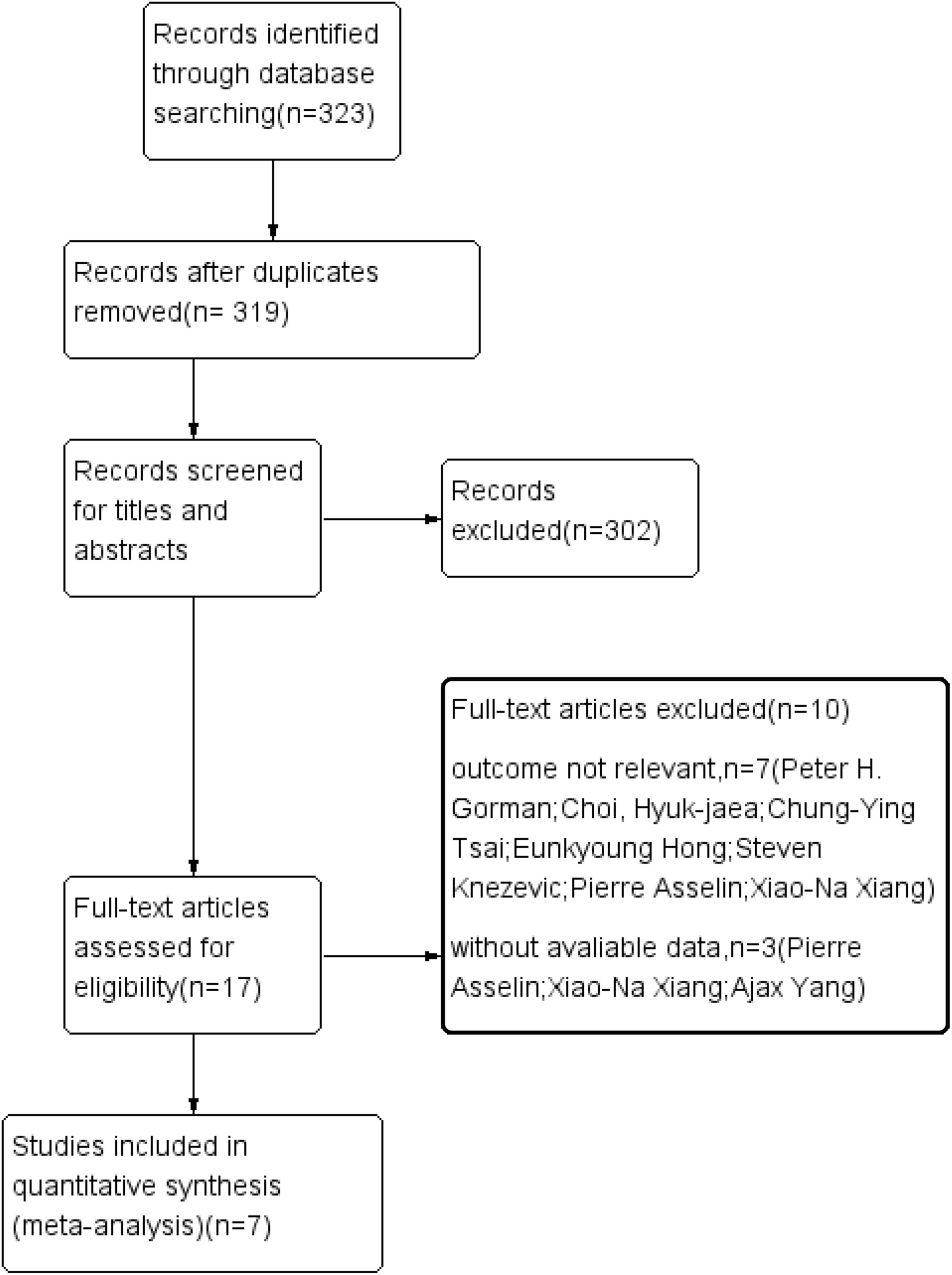
Flow diagram of the literature selection process.

Three electronic databases, namely, PubMed, Embase, and the Cochrane Library, were searched following the Preferred Reporting Items for Systematic Reviews and Meta-Analyses guidelines (14). The final search was conducted in August 2021. We searched all the articles related to patients with SCI receiving Locomat and wearable EAW. The following search terms were used: [“spinal cord injury (MeSH)”] and [“exoskeletal-assisted walking (MeSH)”]. A flowchart of the literature search is GIVEN in Figure 1.

### Inclusion and Exclusion Criteria and Data Extraction

The inclusion criteria for eligible studies were as follows: (1) patients were clinically diagnosed with SCI, (2) patients with SCI were treated with Locomat and wearable EAW, (3) the measurement of intervention was not limited, and (4) at least one outcome of interest was present: 10-MWT, 6-MWT, timed up and go (TUG), and walking index for spinal cord injury (WISCI-II). The exclusion criteria were as follows: (1) reviews, (2) studies with missing data or data that cannot be extracted, (3) animal experiments, and (4) studies with duplicate patient data.

Two investigators independently selected the included studies by reading the title and the abstract of each study. They also extracted data from the included studies. Any discrepancies were resolved by consensus with a third examiner. The data extraction template was used to build an evidence table that includes the following items: author, year, sample size, age, gender, disease duration, equipment, intervention program, and outcomes.

### Statistical Analysis

Reviewer Manager 5.3 was used to calculate the data. Network meta-analysis based on the Bayesian framework was used the R 4.04 software. The mean difference with 95% CI was described using the results of each trial, for estimating the overall effects. Statistical heterogeneity was evaluated using the *I*^2^ statistic. When the heterogeneity (*I*^2^) was <50%, homogeneity was indicated in the studies for inclusion, and a fixed-effects model was used for analysis. However, when heterogeneity (*I*^2^) exceeds 50%, heterogeneity in the included studies was indicated. The random-effects model was used to obtain more reliable results. For all analyses, *P* <0.05 was considered statistically significant. Publication bias was assessed using a funnel plot of treatment effect relative to SD.

### Quality and Bias Risk Assessment

Cochrane collaboration's tool for risk assessment for bias was used for assessing the risk of bias in the included studies (15). Bias risk assessment mainly focused on seven aspects: (1) sequence generation, (2) allocation concealment, (3) participant ignorance, (4) ignorance of result evaluation, (5) incomplete result data, (6) selective result reporting, and (7) other biases.

## Results

**Figure 2:**
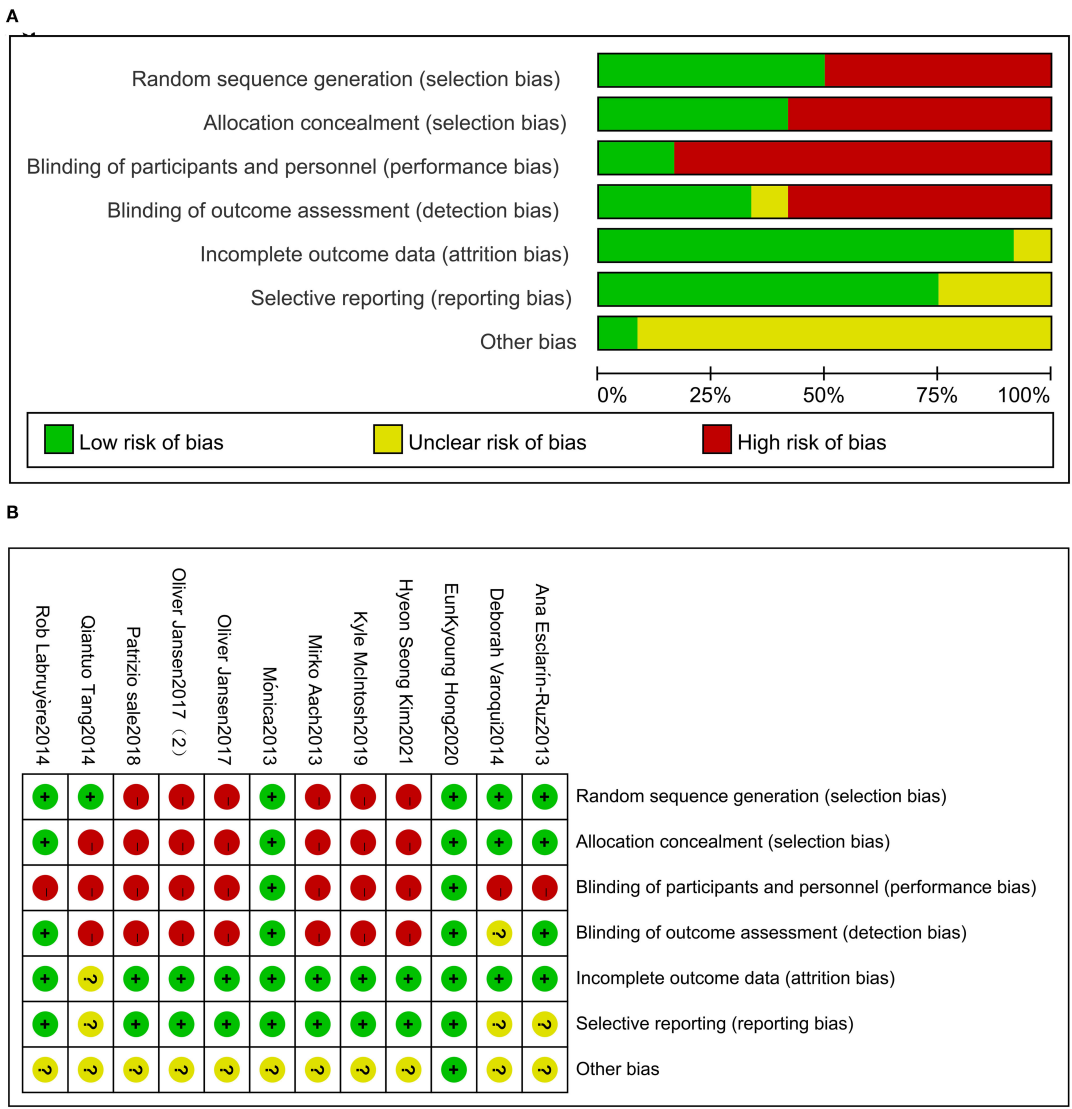
Risk of bias for the included trials **(A)**. Risk of bias summary for the included trials **(B)**.

Of 319 articles initially identified, 297 articles were considered irrelevant based on their titles and abstracts and, thus, were eliminated. The complete data of the 22 studies were further reviewed and 10 studies were subsequently excluded (16–25). The meta-analysis eventually included 12 studies (26–37) containing 353 valid data points on patients with SCI receiving Lokamat and wearable EWA. The publication years of the included articles ranged from 2013 to 2021. A flowchart of the details of the search results and screening process is given in Figure 1. The basic information on the author, year, sample size, age, gender, disease duration, equipment, intervention program, and outcome of the included studies is shown in Table 1. The included studies were evaluated using the risk of a bias graph and summary (Figure 2).

**Table 1:** Characteristic of enrolled studies.

**No**.	**Author**	**Year**	**Sample size**	**Age**		**Gender**	**Equipment**	**Intervention program**	**Patients**	**Outcome**	**Time after injury**	**Neurologic level of injury (NLI)**	**The American Spinal Injury Association Impairment Scale (AIS)**
1	Kim (26)	2021	10	48.1 ± 8.3		7 M/3 F	wearable exoskeleton: H-MEX	60 min of walking training with a powered exoskeleton 3 times per week for 10 weeks (total 30 sessions).	8 were motor-complete SCI (AIS A or B) and 2 were motor-incomplete (AIS C)	6 MWT, TUG	5.68 ± 4.53 (years)	C6, 1; T1, 1; T4, 1; T8, 1; T10, 4; T11, 1; L1, 1.	3.5 ± 0.81
2	Hong (27)	2020	50	38.7 ± 14.2		38 M/12 F	wearable exoskeleton: Ekso+Rewalk	3 times per week for 12 weeks	chronic SCI (6 months)	10 MWT, 6 MWT, TUG	4.69 ± 5.18 (years)	NA	2.74 ± 0.97
3	Mcintosh (28)	2019	11	41 ± 19.8		8 M/3 F	wearable exoskeleton: Ekso GT	25 one-hour sessions of exoskeletal-assisted walking gait training	participants <6 months from initial SCI	10 MWT, 6 MWT	9.55 ± 3.23 (weeks)	C6, 2; T5, 1; T6, 1; T7, 3; T10, 1; T12, 1; L1, 1; L2, 1.	2.82 ± 1.11
4	Sale (29)	2018	8	43.3 ± 12.4		6 M/2 F	wearable exoskeleton: Ekso	20 sessions (5/4 days a week for 4/5 weeks)	7 were motor-complete SCI (AIS A or B) and 1 were motor-incomplete (AIS C)	10 MWT, 6 MWT, TUG	NA	T1, 1; L1, 2; L2, 1; D1, 1; D7, 1; D10, 1; D12, 1.	3.25 ± 0.66
5	Jansen (30)	2017	8	NA		NA	wearable exoskeleton: HAL Robot Suit exoskeleton	12 weeks with 5 training sessions a week.	had acquired SCI more than 1 year prior to enrolment in the trial	10 MWT, 6 MWT, TUG	8.03 ± 6.92 (years)	T 7/8, 1; T12. 2; T11/12, 1; L1, 3; L3, 1.	3 ± 1.12
6	Jansen (31)	2017	21	44.9 ± 13.4		15 M/6 F	wearable exoskeleton: HAL Robot Suit exoskeleton	12 weeks (5 per week; 60 sessions scheduled)	chronic SCI	10 MWT, 6 MWT, TUG	6.49 ± 5.63 (years)	C4, 1; C6, 1; C7, 1; T8, 1; T10, 2; T11, 1; T12, 7; L1, 5; L2, 1; L3, 1.	2.86 ± 1.17
7	Aach (32)	2013	8	47.6 ± 8.8		6 M/2 F	wearable exoskeleton: HAL Robot Suit exoskeleton	90 days period of HAL® exoskeleton (Cyberdyne Inc.) training (5 per week)	in the chronic stage of traumatic 13 spinal cord injury according to time since injury of 1 to 19 years	10 MWT, 6 MWT, TUG	8.03 ± 6.92 (years)	L1, 3; L2, 1; T8, 1; T11, 1; T12, 2.	3 ± 1.12
8	Esclarín-Ruz (36)	2013	88	LKOGT (A1)	43.6 (12)	15 M/6 F	Lokomat	30 min of conventional mobility training plus 30 min of robotic-assisted mobility training	SCI onset (upper motor neuron and lower motor neuro)	10 MWT, 6 MWT	125.6 (65.2) (days)	C1-8, 12;T1-6, 4; T7-11, 5.	1.67 ± 0.47
				LKOGT (B1)	36.4 (12)	14 M/6 F					117.9 (25.6) (days)	T12-L1, 14; L2-3, 6.	1.67 ± 0.47
				OGT (A2)	44.9 (7)	13 M/8 F		60 min of conventional mobility training			140.3 (45.5) (days)	C1-8, 12; T1-6, 4; T7-11, 5.	1.76 ± 0.43
				OGT (B2)	42.7 (18)	17 M/4 F					109 (50.5) (days)	T12-L1, 16; L2-3, 5.	1.76 ± 0.43
9	Alcobendas-Maestro (33)	2012	80	Lokomat	45.2 (15.5)	62 M/38 F	Lokomat	the Lokomat group completed 30-min sessions with the Lokomat in each walking sessio	Within 3–6 Months of Incomplete Spinal Cord Lesion	10 MWT	120 (87.5–145) (days)^#^	C1-8, 22; T1-T6, 7; T7-T12. 8.	1.68 ± 0.47
				Conventional	49.5 (12.8)	63 M/37 F					135 (93.7–180) (days)^#^	C1-8, 23; T1-T6, 5; T7-T12. 10.	1.17 ± 0.50
10	Varoqui (37)	2014	30	Lokomat	50.8 (2.12)	8 M/7 F	Lokomat	Lokomat three times a week over 4 weeks, for a total of twelve training sessions. Each session lasted 1 h, including set-up time, with between 30 and 45 min of training.	chronic SCI	10 MWT, 6 MWT, TUG	11.80 ± 2.54 (years)	C2-7, 11; T1-7, 4.	NA
				Conventional	44.65 (2.66)	14 M/1 F					8.09 ± 1.89 (years)	C2-7, 9; T1-7, 6.	NA
11	Labruyère (35)	2014	9	59 (11)	5 M/4 F	Lokomat	Training duration per session was 45 min for both interventions (actual training time, including maximally 2 breaks of 1–2 min during RAGT, and including warming-up during strength training and breaks to change from one exercise to the next).	Chronic iSCI (time after injury >1 y) and sensorimotor incomplete	10 MWT	50 ± 56 (mo)	C4, 2; C5, 2; C6, 1; T4, 1; T8, 1; T11, 2.	1.5	
12	Tang (34)	2014	30	Lokomat	38.1 (7.1)	30 M	Lokomat	The training time of Lokomat groups was 40 min.	incomplete spinal cord injury	10 MWT	NA	NA	NA
				Ergo_bike	39.2 (8.1)								

*M, male; F, female; SCI, spinal cord inury; MWT, meter walk test; TUG, timed up and go test; H-MEX, Hyundai Medical Exoskeleton; HAL, hybrid assistive limb. #, median (interquartile range)*.

### Wearable EAW for 10-MWT and 6-MWT in Patients With SCI

**Figure 3:**
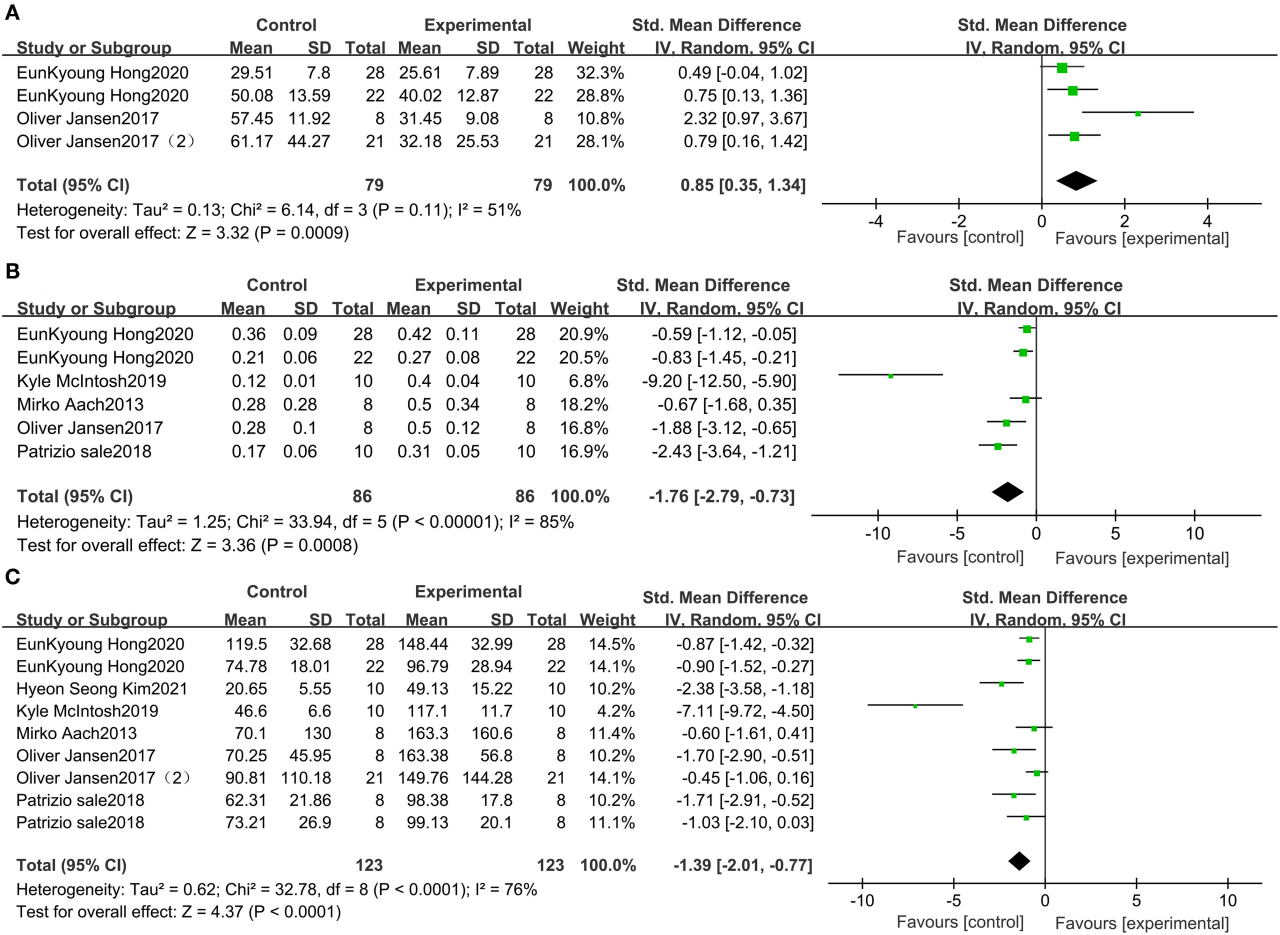
Change from baseline in the distance traveled in the 10-MWT **(A)**, the speed traveled in the 10-MWT **(B)** and the distance traveled in the 6-MWT **(C)**.

This study included 79 patients with SCI from 3 studies that included data on wearable EAW for 10-MWT. The distance traveled in the 10-MWT was significantly improved by wearable EAW [0.85 (95% CI = 0.35, 1.34)] relative to that of the baseline, and the *I*^2^ test for inconsistency was 51% (Figure 3A). A meta-analysis of 5 studies with 86 participants on 10-MWT speed was conducted. The 10-MWT speed score significantly improved by receiving wearable EAW relative to that of the baseline [−1.76 (95% CI = −2.79, −0.73)], and the *I*^2^ test for inconsistency was 85% (Figure 3B). Also included were 123 patients with SCI from 6 studies, with data available on the distance covered during the 6-MWT. The distance traveled in the 6-MWT was significantly improved by receiving wearable EAW relative to that of the baseline [−1.39 (95% CI = −2.01, −0.77)], and the *I*^2^ test for inconsistency was 76% (Figure 3C).

### Wearable EAW for TUG in Patients With SCI

**Figure 4:**
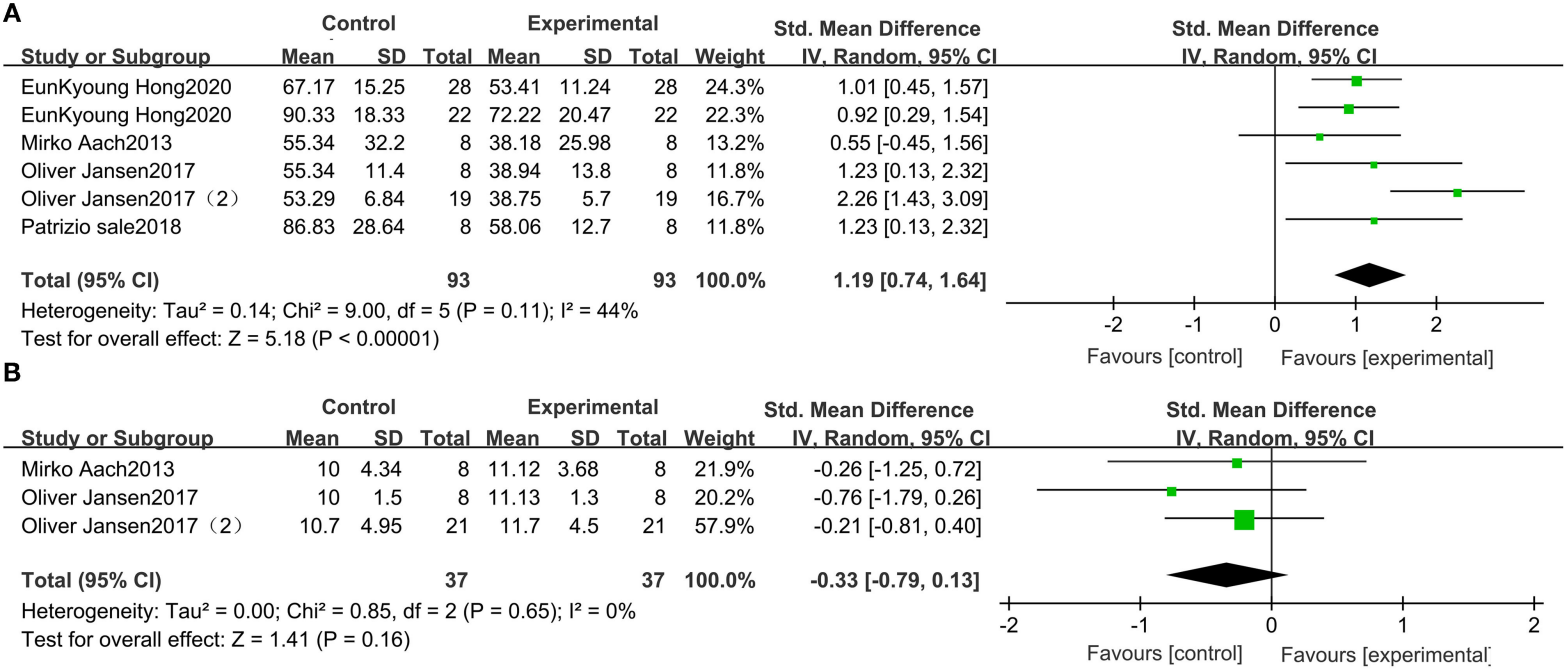
Change from baseline in TUG **(A)** and WISCI-II **(B)**.

Five studies, which included 93 participants, compared the TUG scores of patients with SCI. The TUG scores were significantly improved by receiving wearable EAW, relative to those of the baseline [1.19 (95% CI = 0.74, 1.64)]. Heterogeneity was observed among these groups (*I*^2^ = 44%) (Figure 4A).

### Wearable EAW for the WISCI-II in Patients With SCI

Three studies were included, which comprised 37 patients with SCI whose WISCI-II data were available. No significant increase in the WISCI-II scores [−0.33 (95% CI = −0.79, 0.13)] by receiving wearable EAW was indicated. Heterogeneity was observed among these groups (*I*^2^ = 0%) (Figure 4B).

### Sensitivity Analysis

**Figure 5:**
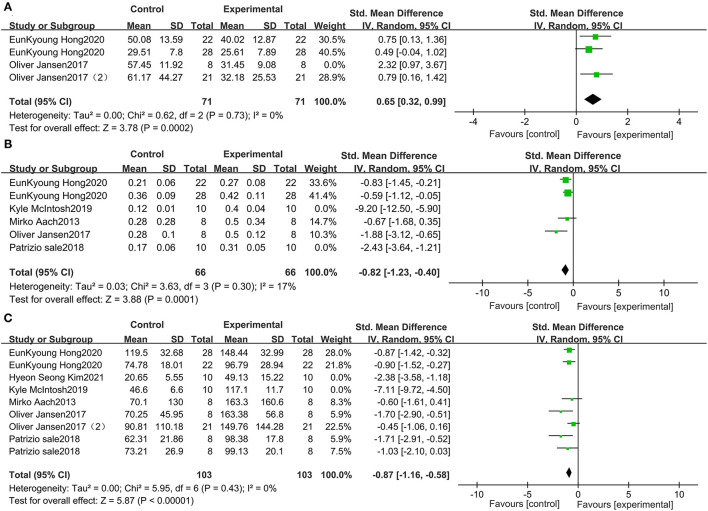
Forest plots for the sensitive analysis. Change from baseline in the distance traveled in the 10-MWT **(A)**, the speed traveled in the 10-MWT **(B)**, and the distance traveled in the 6 MWT **(C)**.

To eliminate heterogeneity in the 10-MWT time, we excluded one study (30) in the meta-analysis. The results indicated that the 10-MWT score was significantly improved by receiving wearable EAW relative to that of the baseline [0.65 (95% CI = 0.32, 0.99)]; heterogeneity was observed among these groups (*I*^2^ = 0%; Figure 5A). To eliminate heterogeneity in the 10-MWT speed, we excluded two studies (28, 29) from the meta-analysis. The result showed that wearable EAW significantly improved the 10-MWT speed scores relative to that of the baseline [−0.82 (95% CI = −1.23, −0.40)] and heterogeneity was observed among these groups (*I*^2^ = 17%; Figure 5B). To eliminate heterogeneity in the 6-MWT distance, we excluded two studies (26, 28) in the meta-analysis. The results showed that the distance covered in the 6-MWT was significantly improved by EAW relative to that of the baseline [−0.87 (95% CI = −1.16, −0.58)] and heterogeneity was observed among these groups (*I*^2^ = 0%; Figure 5C).

### Lokomat for the 10-MWT in Patients With SCI

**Figure 6:**
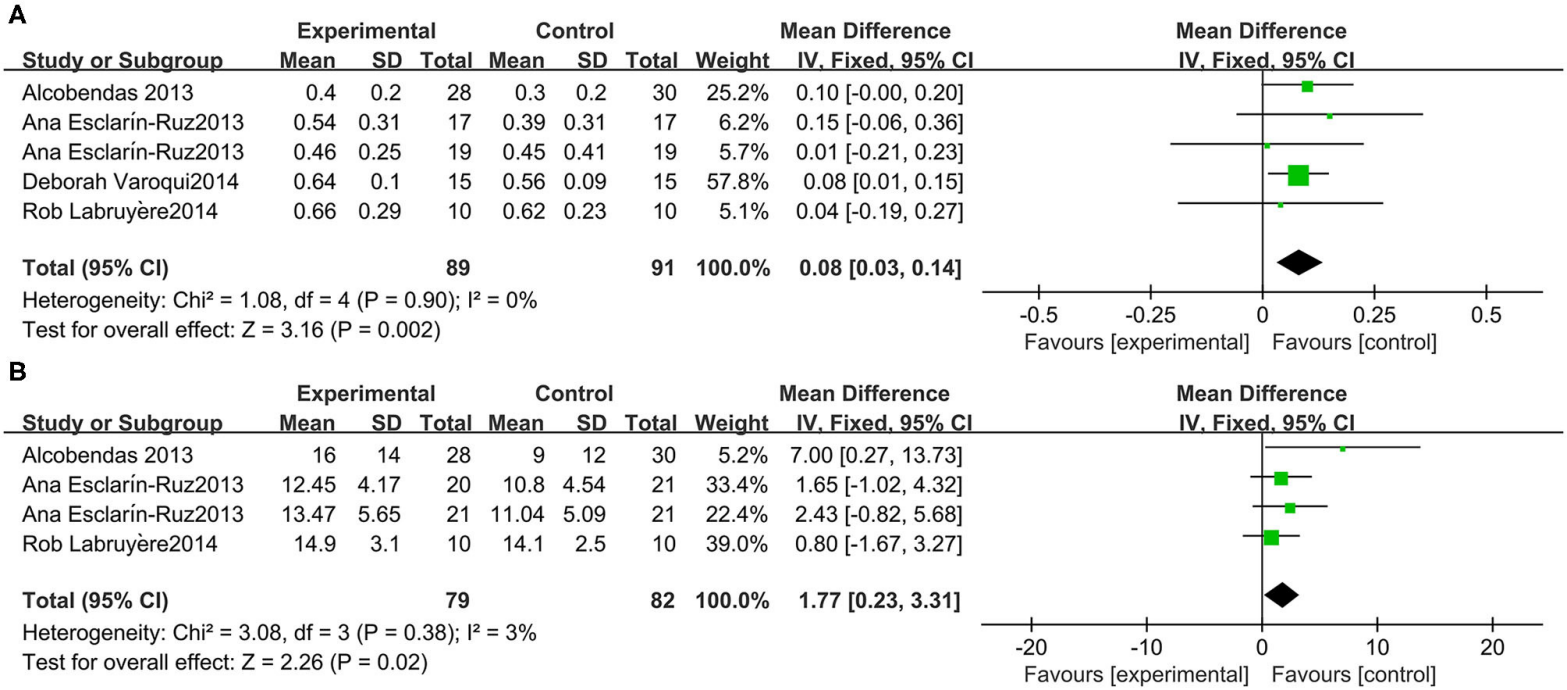
Change from baseline in the speed traveled in the 10-MWT **(A)** and the WISCI-II **(B)**.

A meta-analysis of 3 studies was conducted with 91 participants on 10-MWT speed. The 10-MWT score was significantly improved by Lokomat [−0.08 (95% CI = 0.14, −0.03)] and the *I*^2^ test for inconsistency was 0% (Figure 6A).

### Lokomat WISCI-II in Patients With SCI

Three studies were included, which comprised 82 participants with SCI whose WISCI-II data were available. A significant increase in the WISCI-II score [1.77 (95% CI = 0.23, 3.31)] by Lokomat was indicated. Heterogeneity was observed among these groups (*I*^2^ = 3%) (Figure 6B).

### Network Meta-Analysis

**Figure 7:**
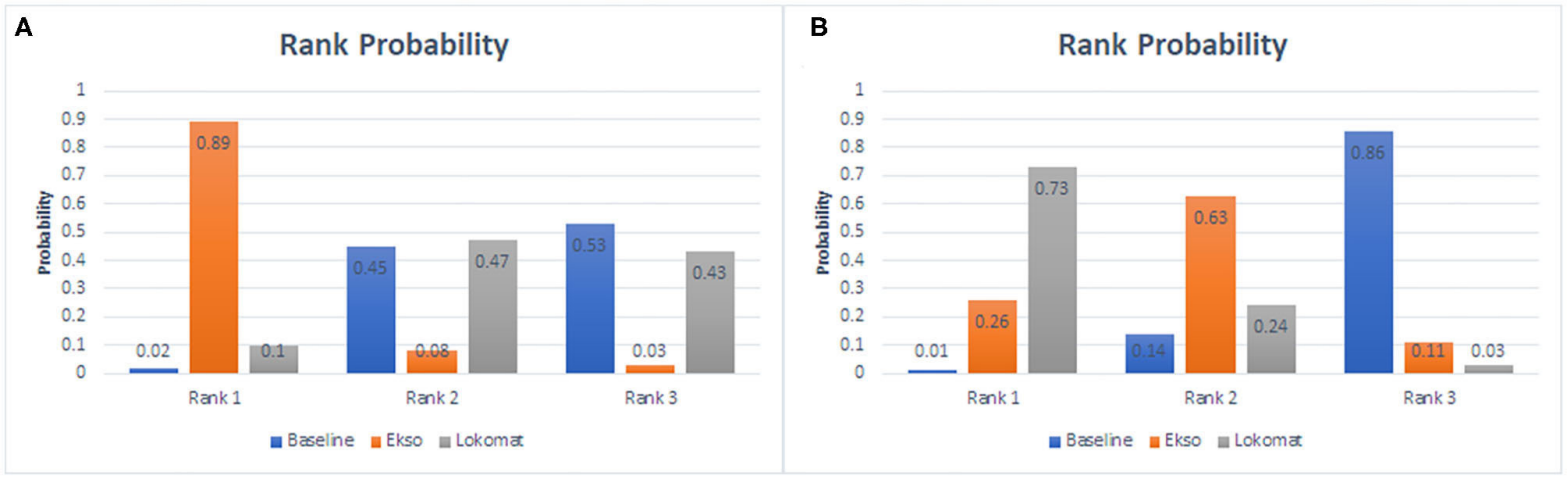
Rank probability of each target strategy by the improvement of wearable EAW and Lokomat on 10-MWT speed **(A)** and the improvement of wearable EAW and Lokomat in the WISCI-II scores **(B)**.

The ranking plot given in Figure 7 shows the probability of each target strategy ranking in terms of efficiency. Network meta-analysis explores the improvement of wearable EAW and Lokomat on 10-MWT speed. The probability of wearable EAW to ranking first was 89% and that of wearable EAW ranking second was 47%. The network meta-analysis explored the improvement of wearable EAW and Lokomat in the WISCI-II scores. The probability of Lokomat to rank first was 73% and that of wearable EAW to rank second was 63%.

### Meta-Regression Analysis

We conducted meta-regression analysis for comparing baseline demographic and clinical characteristics. Our results indicated that age, time after injury, and the American Spinal Injury Association Impairment Scale (AIS) had no impact on the outcomes of patients undergoing wearable EAW and Lokomat training (*P* > 0.05; Supplementary Materials).

## Discussion

Robotic gait rehabilitation emerged nearly 30 years ago. Intense technological development of robotic devices for gait training gained acceleration since the development of Lokomat in 1994 (38). Different rehabilitation robots are mainly classified into grounded exoskeletons (e.g., Lokomat) and wearable exoskeletons (e.g., ReWalk and Ekso). Wearable exoskeletons are increasingly being used for gait rehabilitation. A few have already been approved by the FDA and/or CE mark and are commercially available (i.e., Ekso, HAL, Indego, REX, ReWalk, and SMA) (10). Although several reviews on wearable exoskeletons have been carried out, evidence is still lacking on the effectiveness and usability of exoskeletons for the clinical neurorehabilitation of patients with SCI.

Both the interventions (Lokomat and wearable EAW) in our study have a common goal of regaining or improving locomotion among patients with SCI. As one of the most frequent outcome measures, the ambulation assessment category has been the most used (10). And the gait speed, the 10-MWT, the 6-MWT, and the TUG test as the most important and frequent outcome measures were reported in previous systematic reviews (5, 10). In this study, we conducted 10-MWT, 6-MWT, TUG test, and WISCI-II to evaluate the participant's locomotor ability.

As the clinical characteristics and baseline demographic data of patients selected to undergo the RAGT might influence the efficacy of the interventions, we performed meta-regression analysis for comparing clinical characteristics and the baseline demographic in this study.

For clinical characteristics, our result showed that time after injury and AIS did not affect the outcomes of patients undergoing wearable EAW and Lokomat (*P* > 0.05).

### Participant's Types (AIS)

Certain participants might respond to the intervention better than certain others. The correlation between AIS and gait speed suggested that the participants' type would not influence the outcomes of the interventions. It was difficult to conduct subgroup analyses because of insufficient trials and participants (39). In this study, most participants suffered a complete motor injury in the wearable EAW group and incomplete motor injury in the Lokomat group, indicating that both interventions could benefit the locomotor ability of different types of participants.

### Participant's Neurological Level of SCI and Time After Injury

In this study, the range of level of injury (LOI) covered high cervical levels (C1) to low lumbar lesions (L3). The grounded exoskeletons group (Lokomat) ranged from C1 to L3 and the wearable EAW ranged from C4 to L3 (Table 1). The range of LOIs for wearable exoskeletons seems to be different from earlier reviews that reported the range of LOIs from C3 to L5 (10) or C4 to L4 (9). The most common were patients with thoracic lesions, which may be due to the study inclusion/exclusion criteria. An earlier review (5) reported that higher speeds were associated with a lower LOI when walking with an exoskeleton as an assistive device. They suggested that individuals with better neurological preservation of their spinal cord are more likely to achieve greater speeds. But because of the lack of detailed data on each participant, the correlation between LOIs and locomotor measures cannot be analyzed.

The time since injury might influence the outcomes as it may be correlated to participants' functional presentation. Similar to an earlier review (5), our results also indicated no correlation between time after injury and locomotor measures, which may also be attributed to the study inclusion/exclusion criteria. We conclude from our results that all the participants could benefit from RAGT at different times after injury.

For the baseline demographic, our results showed that age did not affect the outcomes in patients undergoing wearable EAW and Lokomat (*P* > 0.05). However, an earlier review (4) reported a different result, in which they found a significant correlation between increasing age and faster gait speed (*r* = 0.27; *p* = 0.18), attributing it to the epidemiology of SCI among younger individuals with SCI who tend to sustain a traumatic SCI, resulting in a higher LOI. Nevertheless, they found a non-significant correlation between increasing age and lower levels of injury (i.e., less neurological impairment). In our study, age exhibited no effect on the results, and we assumed that all individuals of any age would benefit from both types of RAGT.

Participants either regained or improved their locomotor ability with a reasonable training program. And to our knowledge, the training intensity was highly relevant for participants. Certain studies emphasized the time and effort required by patients with SCI to learn to undergo the wearable EAW training. For instance, Kozlowski et al. (40) and Khan et al. (41) quantified the time and effort required by patients with SCI to learn to use the ReWalk exoskeleton. And the number of training sessions used in earlier studies on overground exoskeletons varies largely from 5 sessions to 60 or more sessions (41); another study (5) reported that an exoskeleton powered for a longer time achieved faster gait speeds. Because SCI is heterogeneous by nature, a few studies reported the details of training sessions, such as the performance of these devices on different terrains and environments. Besides, a majority of previous studies were observational studies, which implies questionable evidence. Randomized control trials are needed to demonstrate that the intensity of training affects the efficacy of RAGT.

Our findings showed that these two types of RAGT could help patients with SCI in improving their locomotor ability. Generally, wearable EAW had positive effects on the locomotor ability of patients with SCI but had relatively high heterogeneity in both 10-MWT and 6-MWT. After eliminating heterogeneity, we found notable group improvements consistent with earlier studies in pre-, mid-, and post-intervention measurements in our study across both the 6-MWT (40, 42, 43) and 10-MWT (43–46). Notable improvements were also found in the TUG test in our study, a commonly used screening tool to identify patients at risk of falling (47). Wearable EAW seemed to be an effective way to help patients with SCI to lower the risk of falling, as evidenced in a few earlier studies (42–44).

As a typical stationary exoskeleton-type device, Lokomat guided the patient's limbs and simulated a symmetrical bilateral gait (48). It has been used to help patients with SCI to regain or improve locomotor ability. Notable improvements by Lokomat in 10-MWT speed were found in our study. As indicators of locomotor ability are valuable clinical effects reflecting physical ability, exoskeletons enabled patients to walk further during the 6-MWT, complete 10-MWT faster, and improve the completion time during the TUG test.

In this study, notable improvements in 10-MWT speed by wearable EAW and Lokomat were observed, but no significant change was found in the WISCI-II of the two types of RAGT. Hence, we assumed that the exoskeleton improved the locomotor abilities of patients with SCI by increasing their walking speed. The ranking of the improvements in 10-MWT speed demonstrated that wearable EAW had a higher probability of being ranked as the better technique followed by Lokomat; therefore, we concluded that wearable EAW yielded better outcomes in locomotor indicators in patients with SCI. This might be due to the difference between a stationary system on a treadmill (Lokomat) and an overground walking system (wearable EAW). Patients need to put more effort with the trunk and arms for mobility while undergoing wearable EAW. Earlier studies showed that compared with Lokomat, Ekso stimulated higher trunk (49) and pelvic floor (50) muscle activity and induced greater changes in the motor and sensory functions (41), which helped patients achieve better mobility by using the torso during walking to enhance the balancing ability, while also demanding higher cognitive and cardiovascular efforts (51). Moreover, 10-MWT was performed on a clear pathway and not on a treadmill. Wearable EAW provided overground walking programs that imparted patients with more proprioception stimulation and were more adaptive to functional daily life environment and testing environment. It also provided more freedom of movement during walking, which activated mechanisms of neuroplasticity and connectivity re-modulation (52), and might promote motor and functional recovery in patients with SCI. To our knowledge, generalization of motor learning could be sensitive to speed (53); therefore, walking faster might improve motor function and promote motor plasticity (54). Based on the importance of walking speed in evaluating locomotor abilities, we concluded that wearable EAW yielded better outcomes in locomotor indicators in patients with SCI.

The ranking of the improvement on the WISCI-II indicated that Lokomat resulted in better improvement than wearable EAW. But no significant changes were found in the WISCI-II of the two types of RAGT in our study, possibly because the WISCI-II was used as a walking capacity scale for patients with SCI who could stand and walk with assistance in clinical trials without evaluating the walking speed. However, a further increase in walking capacity was observed in patients with SCI aided by exoskeletons as determined based on their walking speed. Thus, Ditunno (55) suggested combining the WISCI-II with walking speed measurement to assess the locomotor ability of patients with SCI related to the ceiling effect of the WISCI. Lokomat users exhibited better improvement than wearable EAW users on the WISCI-II, as Lokomat allowed patients to be more focused on their gait and balance (56) in the absence of environmental disturbance compared with the stationary wearable EAW system, which helped people with SCI be more mobile and can independently complete simple tasks in daily life, thus positively enhancing the quality of life of patients (57).

Existing evidence indicates that RAGT contributed to the reduction of secondary health complications in an SCI population and allowed them to walk faster and farther for a longer duration, increased exercise intensity (58), and ultimately applied the physical activity guidelines for health recommended by the WHO (59). We concluded that these two types of RAGT could help patients with SCI to avoid a predominantly sedentary lifestyle (60), diminish secondary health problems, and enhance cardiovascular fitness by improving aerobic capacity (61–63). Compared with the Lokomat, the powered robotic exoskeletons are compact, lightweight, and portable. Wearable EAW seemed to be a more promising training approach and met further requirements of walking capacity improvement in patients with SCI. Under the assumption that wearable EAW could sufficiently yield the aforementioned health benefits, the cost of providing personal wearable EAW to individuals with SCI for home use may be offset by preventing health disorders associated with prolonged sitting. This wearable EAW program may ultimately lead to overall savings in the healthcare system. Further research, especially randomized control trials, is needed to demonstrate the clinical efficacy of wearable EAW, and it is necessary to explore the longitudinal effects of wearable EAW training.

## Limitation

To the best of our knowledge, our network meta-analysis is the first to compare the effects of Lokomat and wearable EAW on individuals with SCI. The limitations of this meta-analysis that limited the inferences of our research are as follows: relatively small number of articles involving non-randomized control trial research articles and the small sample size of most of the articles. Also, further studies need to focus on understanding whether the intensity of training affects the efficacy of RAGT and in addition to the mechanisms by which RAGT improved walking recovery capabilities.

## Conclusion

The results of the network meta-analysis clearly evidenced that these two types of RAGT had positive effects on the performance of locomotion abilities, namely, distance, speed, and function. EAW stimulated a greater muscle activity among participants and prompted higher cognitive efforts, which might lead to better outcomes in walking speed compared with that in the case of Lokomat. As powered robotic exoskeletons are compact, lightweight, and portable, EAW seemed to be a more promising training approach.

## Data Availability Statement

The original contributions presented in the study are included in the article/Supplementary Material, further inquiries can be directed to the corresponding author/s.

## Author Contributions

CC and LS contributed to the conception of the study. LZ and FL contributed significantly to analysis and manuscript preparation. All authors contributed to the article and approved the submitted version.

## Conflict of Interest

The authors declare that the research was conducted in the absence of any commercial or financial relationships that could be construed as a potential conflict of interest.

## Publisher's Note

All claims expressed in this article are solely those of the authors and do not necessarily represent those of their affiliated organizations, or those of the publisher, the editors and the reviewers. Any product that may be evaluated in this article, or claim that may be made by its manufacturer, is not guaranteed or endorsed by the publisher.
